# Interacted QTL Mapping in Partial NCII Design Provides Evidences for Breeding by Design

**DOI:** 10.1371/journal.pone.0121034

**Published:** 2015-03-30

**Authors:** Su Hong Bu, Zhao Xinwang, Can Yi, Jia Wen, Tu Jinxing, Yuan Ming Zhang

**Affiliations:** 1 State Key Laboratory of Crop Genetics and Germplasm Enhancement / Jiangsu Collaborative Innovation Center for Modern Crop Production, Nanjing Agricultural University, Nanjing, Jiangsu, China; 2 Statistical Genomics Lab, College of Plant Science and Technology, Huazhong Agricultural University, Wuhan, Hubei, China; Temasek Life Sciences Laboratory, SINGAPORE

## Abstract

The utilization of heterosis in rice, maize and rapeseed has revolutionized crop production. Although elite hybrid cultivars are mainly derived from the F_1_ crosses between two groups of parents, named NCII mating design, little has been known about the methodology of how interacted effects influence quantitative trait performance in the population. To bridge genetic analysis with hybrid breeding, here we integrated an interacted QTL mapping approach with breeding by design in partial NCII mating design. All the potential main and interacted effects were included in one full model. If the number of the effects is huge, bulked segregant analysis were used to test which effects were associated with the trait. All the selected effects were further shrunk by empirical Bayesian, so significant effects could be identified. A series of Monte Carlo simulations was performed to validate the new method. Furthermore, all the significant effects were used to calculate genotypic values of all the missing F_1_ hybrids, and all these F_1_ phenotypic or genotypic values were used to predict elite parents and parental combinations. Finally, the new method was adopted to dissect the genetic foundation of oil content in 441 rapeseed parents and 284 F_1_ hybrids. As a result, 8 main-effect QTL and 37 interacted QTL were found and used to predict 10 elite restorer lines, 10 elite sterile lines and 10 elite parental crosses. Similar results across various methods and in previous studies and a high correlation coefficient (0.76) between the predicted and observed phenotypes validated the proposed method in this study.

## Introduction

Genetic mating design plays an important role in crop genetics and breeding. In hybrid breeding, this design was widely used to evaluate general combining ability of parents and specific combining ability of the F_1_ between two parents. Therefore, many elite hybrid cultivars were bred and utilized in crop production. In classical quantitative genetics, mating design is one of main components. It provided a lot of information about two-order genetic parameters for quantitative traits. However, only the collective effects of all the polygenes were estimated. The introduction of molecular markers has facilitated the mapping of quantitative trait loci (QTL) in numerous species, and substantial progress has been achieved in bi-parental segregation population but not in the mating design. As we know, North Carolina (NC) design II has been considered to be one of the most powerful mating designs for combining ability and heterosis analyses, and its application has expanded into many crops. Therefore, there is a critical need for in-depth study of the methodology for mapping QTL in this design.

During the past several decades, many attempts have been made to detect QTL for quantitative traits in bi-parental segregation populations. For example, single marker analysis [1], two-marker analysis [2], interval mapping [3], composite interval mapping [4,5], multiple interval mapping [6], Bayesian method [7,8], and Bayesian-based likelihood approach [9–11]. However, this bi-parental segregation population is rarely used alone in commercial breeding, and therefore the results from these single-cross experiments have limited roles in breeding practice [12]. To overcome this shortcoming, crop cultivar population was used to conduct genome-wide association study (GWAS) [13,14], and the main and interacted effects of detected QTL were used to carry out breeding by design [14–16]. However, this approach is useful only for inbred line breeding but not for hybrid breeding, because only additive and additive-by-additive (*aa*) effects [14] but not dominant-related effects [17–21] were estimated in the above GWAS.

Up to now many mating designs have been proposed, such as four-way cross [22], triple testcross (TTC) [23], diallel design [24,25], and NC mating design [26]. Current studies on the topic focus mainly on three aspects. The first is the classical genetic analysis [27–29], for example, combining ability analysis [30]. These analyses dissected the genetic foundation of quantitative traits, which provided useful information for crop breeding. However, the position and effect of individual QTL are unclear. To answer this question, the QTL mapping approach is available, which is the second main area of the work on the mating design. In four-way cross, He et al. [12] extended the main-effect QTL mapping of Xu [31] into the interacted QTL mapping. In TTC, *Z*_1_, *Z*_2_ and *Z*_3_ were used to detect augmented additive, augmented dominant and dominance-by-additive (*da*) interaction effects, respectively, in Kusterer et al. [32] and Melchinger et al. [33], and to unbiasedly estimate all the main and epistatic effects in He et al. [34]. In the NCIII, pair mean *Z*_1_ and pair difference *Z*_2_ were used to detect augmented additive effect and augmented dominant effect in Melchinger et al. [35], and epistasis in Garcia et al. [36] and He et al. [37]. In addition, Rebaϊ & Goffinet [38] and Lenarcic et al. [39] developed a general regression-based method and Bayesian approach, respectively, for QTL detection in diallel design; Li et al. [40] and Wang et al. [41] proposed analysis of variance approach for the detection of main and interacted QTL of quantitative and endosperm traits in NCIII and TTC, respectively; and Reif et al. [42] used TTC with near-isogenic lines as base population to detect epistasis. However, relatively little has been known about NCII mating design. Recently, different base populations along with several testers were used to dissect the genetic foundation of heterosis using QTL mapping of GCA and SCA, such as BC_1_F_8_ [43], RIL [44] and introgression lines [45,46]. More recently, the comparison across different base populations was conducted as well [47]. However, these studies are based on main-effect QTL model. Finally, the mating design has been adopted in crop breeding, for example, four-way cross in maize breeding, and NCII mating design in rice and maize hybrid breeding. However, genetic analysis and crop breeding have been often performed separately.

In hybrid breeding, one group of parents is crossed with another group of parents, namely NCII mating design, in order to select elite hybrid cross. However, partial (or unbalanced) crosses are often conducted in breeding practice. In this study, partial F_1_ hybrids along with their parents, a partial NCII mating design, were used to conduct genetic analysis. Here all the main and interacted effects were included in one full model. Two-dimension interacted effects between each pair of QTL were considered as interacted term. If the number of effects in the model was 10 times more than sample size, two groups of extreme individuals were used to test which effects were related to the trait. All the selected effects were further shrunk by empirical Bayesian, so significant effects could be identified. A series of Monte Carlo simulations was performed to validate the new method. The validated approach was used to dissect the genetic basis of oil content in rapeseed in partial NCII mating design. Based on the above information, novel parents and cross combinations would be predicted.

## Results

### Effect of QTL heritability on mapping QTL

**Fig 1:**
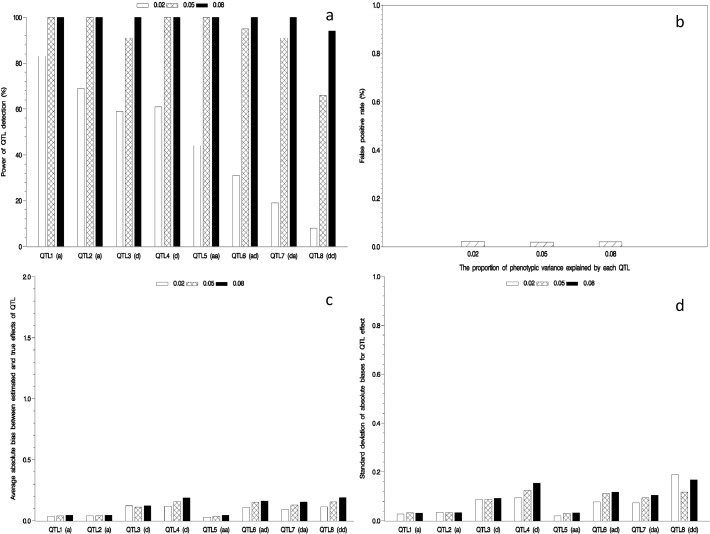
Effect of QTL heritability on mapping QTL in the NCII. Power of QTL detection (a); false positive rate (b); and average (c) and standard deviation (d) of absolute bias between estimated and true effects.

In the first simulation experiment, the effect of QTL heritability on QTL mapping in the NCII population was evaluated by letting QTL heritability be set as 0.02, 0.05 and 0.08. Note that the number of effects in the full model is 84,050, which is 116 times more than sample size. At this situation, high throughput QTL-effect screening approach described in this study was adopted. The results are shown in Fig. 1 and Supporting Information S1 Table. A general trend was found. In other words, the power of QTL detection increases as QTL heritability increases. Relatively small estimates for the average and standard deviation of absolute bias between estimated and true effects as well as false positive rate (FPR) were observed in the above three situations. In the same QTL heritability, the power is higher for main-effect QTL than for interacted QTL. In addition, the lowest power in the detection of the dominant-by-dominant (*dd*) interaction is observed in all the situations. The reason may be due to the low proportion of heterozygous genotypes in the mapping population.

### Effect of sample size on mapping QTL

**Fig 2:**
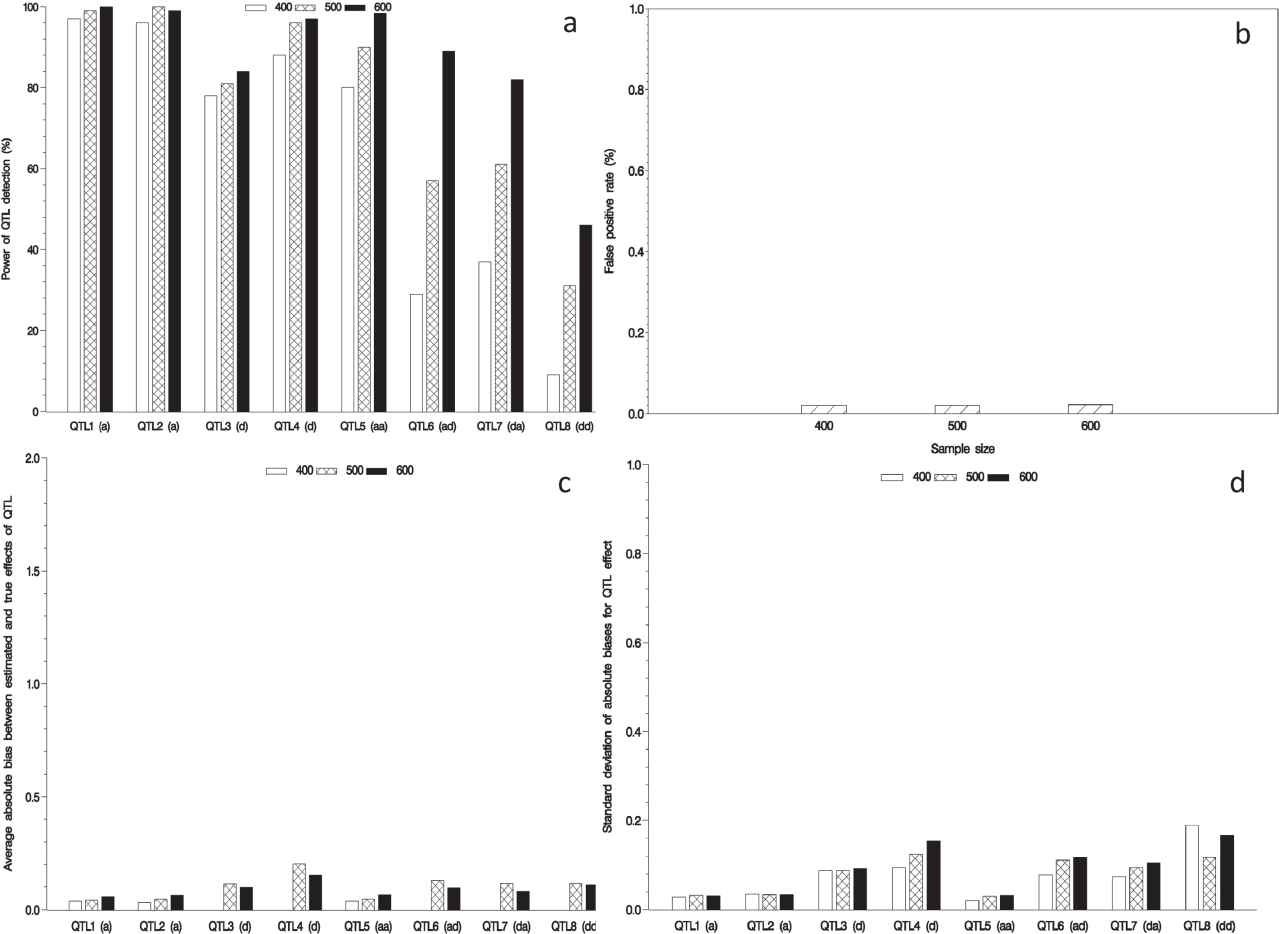
Effect of sample size on mapping QTL in the NCII. Power of QTL detection (a); false positive rate (b); and average (c) and standard deviation (d) of absolute bias between estimated and true effects.

In the second simulation experiment, we explored the effect of sample size on QTL mapping by letting sample size be set as 400, 500 and 600. Note that the proportion of the paternal lines, maternal lines and their hybrids was set at 1:1:2. The others were the same as those in the first simulation experiment. The results are shown in Fig. 2 and Supporting Information S2 Table. A general trend was also found, for example, the power of QTL detection increases as sample size increases. Relatively small estimates for the average and standard deviation of absolute bias between estimated and true effects as well as the FPR were found in the above three situations.

### Effect of population structure on mapping QTL

**Fig 3:**
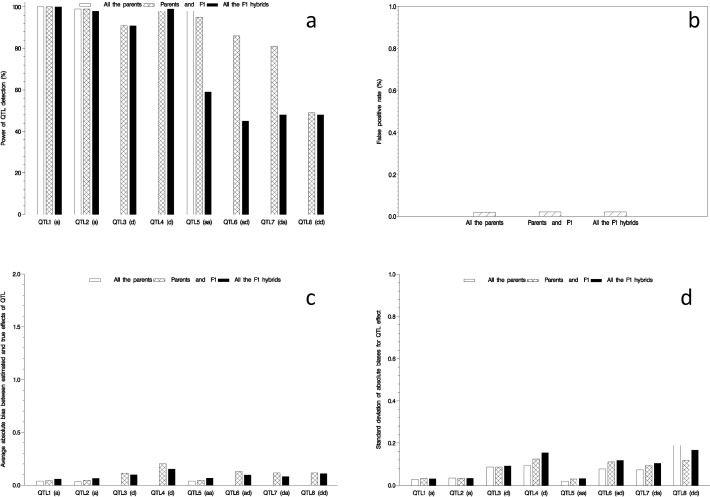
Effect of population structure on mapping QTL in the NCII. Power of QTL detection (a); false positive rate (b); and average (c) and standard deviation (d) of absolute bias between estimated and true effects.

In the third simulation experiment, we investigated the effect of population structure on QTL mapping. The results are shown in Fig. 3 and Supporting Information S3 Table. If all the parents were viewed as mapping population, only additive and *aa* effects could be detected. It is reasonable. This is because only homozygous genotypes were included in the mapping population. If all the F_1_ hybrids were viewed as mapping population, additive and dominant effects could be identified with satisfactory powers. Although all kinds of interacted effects could be detected, their powers were not high, with the highest power for *aa* effect and with the lowest power for *dd* effect. Meanwhile, the average and standard deviation of absolute bias between estimated and true effects were larger for dominant-related effects than for additive and *aa* effects. If parents and F_1_ hybrid were mixed with the same proportion, their powers in the detection of *aa*, additive-by-dominant (*ad*) and *da* effects were significantly higher than those in the only F_1_ hybrids.

### Mapping QTL for oil content in *Brassica napus*

One *Brassica napus* breeding dataset from Professor Jinxing Tu at Huazhong Agricultural University was used for the further demonstration. The dataset was collected from a partial NCII breeding design that contained 298 sterile lines, 143 restorer lines and their 284 F_1_ hybrids. The phenotype analyzed was oil content. A total of 205 markers were used in the analysis. The total number of effects included in the genetic model is 84,050. Therefore, high throughput QTL-effect screening approach described in this study was adopted.

In the first step, 72 parental lines or F_1_ hybrids with the highest oil content and 72 parental lines or F_1_ hybrids with the lowest oil content were selected from the mapping population. Using the two groups of individuals, *χ* ^2^ test of independence was carried out to identify whether one marker under consideration was associated with the trait. In the second step, all the markers associated with the trait were included in the genetic model of the full mapping population. Therefore, association mapping could be conducted. All the results were listed in Table 1.

**Table 1:** Mapping QTL for rapeseed oil content in partial NCII mating design.

QTL	Type	Position (marker)	Allelic frequency		Bulked segregation analysis		Association mapping			Similar results with other methods				Similar results in previous studies
			Locus 1	Locus 2	*χ* ^2^	P-value	LOD	Effect	r^2^(%)	EBLASSO	GEMMA	Regression-based	CV	
1	a	CB10597C	0.54		17.46	0.0002	3.79	0.2619	1.19	√		√	√	Ra2E04~S13M08-1-70[56];e8m17-309~Ra3H09[51]
2	A	Bo3b	0.40		15.52	0.0004	3.13	−0.2680	1.10	√		√	√	
3	a	xy2b	0.73		13.40	0.0012	11.39	0.5597	3.99	√		√	√	
4	a	BnGMS488A	0.86		11.01	0.0041	9.27	0.5831	2.77	√	√	√	√	
5	a	BRAS078A	0.05		8.82	0.0122	14.04	−1.2047	4.12	√	√	√	√	BRAS078A[50]; e8m17-309 ~ Ra3H09 [51];AgCan20a ~ Bras026 [52]
6	a	Ol10-C01	0.84		7.72	0.0211	7.60	−0.4916	2.22	√	√	√	√	HMR612a ~ HMR612b [53]; E7M5c ~ sN0240A [50]
7	a	Bo2d	0.64		6.68	0.0354	4.00	−0.2852	1.28				√	
8	d	CB10597C	0.54		4.43	0.0352	3.10	−0.5672	1.07	√		√	√	
9	aa	CN64c×SA63a	0.26	0.32	18.42	0.0001	6.28	−0.3402	1.77		√	√	√	HMR300c ~ MR133.2 [53]
10	aa	BRAS063a×Na10-C06A	0.29	0.62	12.68	0.0018	2.23	−0.2095	0.68		*	√	√	Bras063a [52]; EM16/EM17c~sN12353b [54]; GIFLP106 [57];Na10-C06[51]
11	aa	Bo3a×CB10288B	0.61	0.43	12.43	0.0020	7.25	0.3888	2.10			*	√	HMR403b~MR229 [53]
12	aa	BnGMS175A×Ra2-G08A	0.59	0.25	12.09	0.0024	4.88	−0.3124	1.42			√	√	
13	ad	CB10431A×CB10234A	0.82	0.71	11.86	0.0027	3.48	0.6691	1.22					IGF9014c~pw179b [56]; CB10431[52];CB10234[52];CB10234~ZAAS763[50]
14	aa	20-1c×Ra3-E05D	0.34	0.17	11.49	0.0032	3.57	0.2579	1.01		*	*		ODD20/GB2-238~EM1/PM4-400 [55]
15	aa	BRAS014B×Na14-H11B	0.46	0.85	11.18	0.0037	4.14	0.2708	1.10			√	√	IGF9014c~pw179b [56]; PM88/PM34-484~SA7/PM56-466 [55];Bras115~W09.CD1 [52]; O|10F04~B0SF1574 [57]; Na14H11[57]; CN32a~E7M5f [50];
16	da	CB10139B×BnGMS3B	0.39	0.83	10.80	0.0045	4.20	0.7397	1.01				√	SF19775 [57]; PM88/PM34-378~CB10139-176 [55]
17	aa	CB10229A×Ra3-E05A	0.63	0.50	10.70	0.0048	6.79	−0.3869	2.15				√	snap1200~GIFzip47a [57]; SA89~Na12C01a [51]
18	aa	CB10036B×CB10343A	0.25	0.75	12.29	0.0021	3.73	0.2794	1.18			*		IGF5154c~pX141eE[56];AgCan9~Bras102b[52];EM2/ME14a~Ra2-A01 [54]; CZ1b705119~CZ1b684396 [52]
19	aa	CN75b×CB10373B	0.67	0.51	12.28	0.0022	5.46	0.3431	1.61				√	HMR438a~HMR310[53]
20	aa	CN1b×Ra2-E12B	0.67	0.64	10.83	0.0045	6.46	−0.3794	1.87			*	√	EM1/BG1-405~SA7/PM63-298 [55]
21	aa	CN64d×20-1b	0.58	0.46	10.65	0.0049	3.62	0.2597	1.00			*		
22	aa	Ol12-D05B×BnGMS385B	0.60	0.82	10.55	0.0051	3.49	−0.3293	1.07			*		e18m6-189~CB10028 [51]; E32M48.255~CB10179 [52];niab013~SF25867[57]
23	aa	MR119d×Na12-A02B	0.46	0.56	10.16	0.0062	5.16	−0.3079	1.44	√	√	√	√	ME16/EM17c~sN12353b[54];Na12-A02[51]
24	da	E5a×Na14-H11A	0.46	0.74	9.19	0.0101	2.56	0.8083	0.76			*		Na14-H11[57];MR216a~MR144[52]
25	ad	xy2b×CB10343B	0.40	0.50	9.19	0.0101	7.35	1.7274	1.83				√	
26	aa	MR097×Ol12-F02B	0.81	0.65	9.03	0.0110	5.66	0.3232	1.65			√		E46M64g~Bras089[52];B087P06-1~SA89[51];Ol12-F02A~Mr216b[52];niab028~FITO516c[51]
27	aa	CB10493C×BnGMS340B	0.15	0.46	8.78	0.0124	3.05	0.2502	0.96		√	*	√	sORG49a[50]
28	da	CN64d×Ol11-C02A	0.33	0.80	8.50	0.0143	3.05	−0.6749	0.98		*	*		IGF9014c~E2HM32-320[56]
29	aa	32_1a×Na10-C06B	0.82	0.54	11.58	0.0031	4.61	0.2997	1.37			*		ODD20/PM16-97~ME2/PM45-384 [55];e4m5-260~CB10632 [51]; ZAAS815b~ZAAS893 [50]
30	dd	Ra2E12×CB10065B	0.22	0.90	9.60	0.0019	3.00	1.1079	0.78					Ra2E12 [56]; FITO131 [51];E38M621~AgCan50 [52];CB10530a~EM9/ME37a [54];CB10065[52];BoGMS1025~SF17359[57]
31	aa	BnGMS352B×Ra3-E05C	0.87	0.47	9.18	0.0102	2.63	−0.2047	0.67		*			e18m6-189~e18m5-374[51];ODD20/GB2-238~EM1/PM4-400[55]
32	da	CB10045A×Ra3-E05B	0.22	0.15	9.01	0.0111	4.09	−0.8761	1.39					E2M3/g~EM11/Me23a [54]; E42M50.55~E41M50.206 [52];sORG49a[50]
33	dd	Bo2d×BnGMS175A	0.62	0.43	9.00	0.0027	2.13	−0.5142	0.52		*, *	*		
34	aa	Bo3a×CB10065A	0.68	0.05	12.45	0.0020	2.65	0.2238	0.77		*	*		CB10065 [52]; BoGMS1025~BrSF50-42 [57];Na12G11~SN12508 [52]; R04.1840~R06.1360 [52]
35	aa	BRMS-036c×CB10364B	0.13	0.96	11.40	0.0033	3.35	−0.2488	0.95		√	*		BRMS036[50];CB10364**[54];H004I05-1~BnGMS312[51]
36	ad	CB10493C×BnGMS3B	0.94	0.61	9.60	0.0082	2.41	0.6348	0.96			*		IGF2021e~S10M03-1-360[56];e10m22-313~CB10028[51]
37	da	CN46b×CB10597C	0.29	0.46	8.00	0.0183	2.89	0.7535	0.81					Ra2E04~S13M08-1-70[56];e8m17-309~Ra3H09[51]
38	aa	BnGMS103B×CB10277B	0.48	0.62	8.84	0.0120	3.51	−0.2578	1.12			*, *		CB10277[54]
39	dd	Ra2-E12A×Ol11-C02A	0.85	0.14	8.47	0.0036	3.35	−0.9321	1.09					RA2E12[56];E38M621~Na12B05[52];IGF9014c~E2HM32-320[56]
40	aa	MD21a×Ol12-D05A	0.69	0.22	8.11	0.0173	3.62	−0.4304	0.96				√	BrBAC138~GIFLP106[57];E32M48.255~SN11670[52];BRAS031a~E2M3e[50]
41	aa	BRAS063a×CB10139B	0.73	0.67	11.89	0.0026	4.58	−0.2801	1.20		√	*	√	
42	aa	CN64d×CN59a	0.08	0.22	8.66	0.0132	2.55	−0.2134	0.68					
43	aa	xy2a×20-1c	0.25	0.86	12.32	0.0021	2.30	0.2178	0.71			*		
44	aa	CN63b×Na12-A02C	0.27	0.68	8.47	0.0145	2.68	−0.2385	0.76			*		Na12-A02[51]
45	da	20-1b×CB10026C	0.65	0.80	12.22	0.0022	2.15	0.5195	0.74					CN32a~E7M5f[50];I20.760~D20.760[52];sN3761b~sR6293b[56]

*a*: additive; *d*: dominant; *aa*: additive-by-additive; *ad*: additive-by-dominant; *da*: dominant-by-additive; *dd*: dominant-by-dominant; r^2^: the proportion of total phenotypic variance explained by a single QTL.

EBLASSO: Fast empirical Bayesian LASSO [11];GEMMA: genome-wide efficient mixed-model association study [48]; Regression-based: Regression-based association study [49]; CV: cross validation;

√: same QTL was detected by other methods;

*: locus linked to the detected locus was identified by other methods.

8 main-effect QTL and 37 interactedQTL were found to be associated with oil content, and explained 17.74% and 42.27% of phenotypic variance, respectively. Of these QTL, the proportion of phenotypic variance explained by each QTL varied from 0.52% to 4.12%, the LOD score varied from 2.13 to 14.04, and most QTL were additive (7 QTL) or *aa* (25 QTL). A few dominant-related QTL might be due to the low proportion (only 17.99%) of heterozygous genotypes in the mapping population. Correlation coefficient of 0.76 between the estimated genotypic value and the observation supported the proposed approach in this study.

To further confirm the above results, three other approaches, including EBLASSO [11], genome-wide efficient mixed-model association study [48] and regression-based association study [49], were used to re-analyze this dataset. All the results were showed in Table 1. Among all the main-effect QTL, three were identified simultaneously by all the four methods and seven were detected by at least three approaches. Among all the interacted QTL, one was detected simultaneously by all the four methods, eight were identified by at least two approaches; and 14 were partially confirmed because one same locus and two linked loci associated with these interacted QTL were found.

More importantly, some similar results were observed in previous studies (Table 1). One marker linked to main-effect QTL and 16 markers linked to the interacted QTL in this study were same as those in previous studies, and three markers linked to main-effect QTL and 32 markers linked to the interacted QTL were close to those in previous studies [50–57]. For example, additive QTL around marker BRAS078A was consistent with that in Zhao et al. [50], Wang et al. [51] and Delourme et al. [52].

Using the above mapping results, the genotypic values for all the missing F_1_ hybrids could be predicted. These predicted values were further used to calculate general combining ability (GCA) and specific combining ability (SCA). Based on these estimates, novel parents and elite F_1_ hybrids could be predicted (Table 2). Note that novel restorer line R092 could produce elite hybrid crosses: B1341 × R092, B0984 × R092, B0641 × R092, B0857 × R092 and B0066 × R092.

**Table 2:** Elite restorer and sterile lines and hybrid combinations.

Elite restorer line		Elite sterile line		Elite hybrid combination		
ID	GCA	ID	GCA	ID	BV	SCA
R092	3.71	B0393	1.67	R092×B1341	49.32	1.69
R587	2.03	B1053	1.66	R465×B0680	48.52	3.32
R552	1.73	B1341	1.65	R092×B0984	48.30	0.80
R110	1.71	B416	1.62	R552×B338	48.20	2.63
R002	1.61	B338	1.57	R092×B0641	48.16	1.99
R0446	1.50	B0680	1.54	R552×B1358	48.03	3.35
R516	1.40	B1308	1.53	R092×B0857	47.99	2.00
R465	1.38	B0984	1.52	R002×B0685	47.88	3.40
R627	1.34	B0552	1.50	R092×B0066	47.88	2.14

## Discussion

There have been several advantages for the current study. First, one interacted QTL mapping approach for quantitative traits in partial NCII mating design had been proposed in this study. Most genetic analyses of previous studies in the designs are combining ability analysis in the polygenic system and little has been known about detecting individual QTL. Then, interaction genetic analysis in this study had been integrated with crop breeding. This overcomes the shortcoming that genetic analysis in bi-parental segregation population has limited roles in breeding practice [12]. Although some similar studies have been reported [13–15,58], most previous studies are for inbred line breeding. As for the prediction of hybrid performance, to use multiple parents and their progenies as genetic population is a good strategy. However, a single marker analysis in Schrag et al. [30,59] and progenies of F_2_-derived lines in Windhausen et al. [60] were adopted. Clearly, new development appeared in this study. Third, how to estimate a large of parameters in oversaturated genetic model had been considered. In this study, the number of effects in the genetic model is 116 times than sample size. To solve this issue, bulked segregant analysis (BSA) along with empirical Bayesian method were used to estimate all the parameters. This approach was confirmed to be feasible in both Monte Carlo simulation studies and real data analysis. Although high proportion of phenotypic variation was contributed by interaction terms in real data analysis, main and interacted QTL could be clearly identified across various approaches and various groups, and the QTL detection power was larger for main QTL than for interacted QTL in a series of simulation experiments. Actually, this high proportion phenomenon was also observed in Mackay [61]. Meanwhile, 14 of 37 interacted QTL were frequently identified in a series of cross validation experiments, and these interacted QTL were similar to the commonly interacted QTL across four approaches in this study (Table 1), although prediction accuracy in cross validation needs to be addressed. To improve the accuracy, genome-wide prediction may be available in the future project. Finally, the new approach might be extended into other mating designs, i.e., unbalanced or balanced factorial crosses, NCI design, diallel crosses and recurrent breeding population, although the new method was designed for partial NCII mating design. In recurrent breeding population, there were heterozygous genotypes in the parents so that the F_1_ hybrid might be a mixture of multiple genotypes. At this situation, family average idea in the widely-used F_2:3_ design [62] was available.

Combining ability analysis has been found to be an effective method in crop breeding. When NCII mating design was completely carried out, it is easy to calculate general combining ability (GCA) and specific combining ability (SCA). At this situation, novel parents and elite hybrid crosses could be easily predicted. These results could be used to direct crop breeding practice. In crop breeding practice, however, partial NCII mating design (unbalanced factorial crosses) is frequently conducted. At this case, how to estimate combining ability is pending, although a mixed linear model approach for phenotypic values was adopted in Schrag et al. [30,59]. In this study, we proposed one method to deal with this issue. That is, the information from the interacted QTL mapping was used to estimate the genotypic values for all the missing F_1_ hybrids, so elite parental combination could be found. Furthermore, GCA could be estimated and favorable parents could be predicted [63,64]. If all the effects with non-zero estimates were used to predict the genotypic values for all the missing F_1_ hybrids, this is similar to genome-wide selection [65–68].

The above genetic analysis was for a single trait. In crop breeding, multiple traits would be improved simultaneously. At this case, we needed to pyramid favorable alleles of all the detected QTL for multiple traits. For example, if oil content, thioglycoside, erucidic acid, yield per plant and thousand kernel weight were considered simultaneously in the real data analysis. Of 268 detected QTL for the five traits (data not shown), B1348 and R484 were found to have 199 and 204 favorable alleles, respectively. These two sterile restorer lines might be considered in crop breeding practice.

If the number of effects in a genetic model is much larger than sample size, it is difficult to estimate these effects. Although at present there have been some methods available, for example, Bayesian shrinkage estimation [7], LASSO [69,70], penalized maximum likelihood [9], empirical Bayesian [10], EBLASSO [11] and empirical Bayesian elastic net [71], these methods are widely used in bi-parental segregation populations but not in genetic mating population. In this study, one biological approach, named BSA or DNA pooling, was used to choose effects that are associated with the trait of interest. Although BSA was proposed in bi-parental segregation populations [72], this analysis was also useful in large scale association studies [73,74]. When considering interacted QTL in genetic model, main and interacted QTL could be clearly identified. In this study, therefore, this analysis along with the test of independence makes most effects be excluded from the full genetic model, and the reduced model is estimable. This approach in this study is an alternative way in the parameter estimation of oversaturated genetic model.

In quantitative genetics, epistasis refers to any statistical interaction between genotypes at two (or more) loci [61]. In our study, *aa*, *ad*, *da* and *dd* interactions are actually statistical terms rather than epistasis, such as defined by Cockerham’s model [75], although some mathematical relationships exist [76]. In F_2_ population, Kao & Zeng [77] gave a very classic example for mapping epistasis, in which orthogonality between the main effect and epistasis was emphasized, because orthogonality between main effect and epistasis may be important for statistical clarification and interpretation. Note that this orthogonality depends on allelic frequency in the studied population [61]. However, only statistical interaction was considered in this study.

## Materials and Methods

### Mapping population and trait evaluation

In NCII mating design, all sterile lines (298) in *Brassica napus* need to be crossed to all restorer lines (143). However, it is infeasible in breeding practice. Here only 284 F_1_ hybrids were conducted at Huazhong Agricultural University (Wuhan, China). In other words, each of 143 restorer lines was crossed to a pair of sterile lines to produce 284 hybrids. Therefore, the mapping population was a partial NCII mating design (unbalanced factorial crosses), including 298 sterile lines, 143 restorer lines and their 284 F_1_ hybrids. Seed oil contents for each parent and F_1_ hybrid were measured by near infrared reflectance spectroscopy, for technical detail the reader was referred to the original study of Tillmann [78].

### SSR markers

205 SSR primer pairs were examined to screen for polymorphisms among all the 441 parents and the genotypes of all the F_1_ hybrids were deduced from their parents. SSR primers were from Chen et al. [79] and Delourme et al. [52], and primer sequences were obtained from http://www.brassica.info/ssr/SSRinfo.htm (prefixed by Ra, Ol, Na, BN, MB, BRMS- and MR) and http://www.ukcrop.net/perl/ace/search/BrassicaDB [80]. Primer pairs prefixed ‘‘BRAS’’ and ‘‘CB’’ were from the electronic supplementary material of Piquemal et al. [81], and those prefixed ‘‘s’’ were obtained from Agriculture and Agri-Food Canada (http://www.brassica.agr.gc.ca/index_e.shtml). PCR experiment was described in Chen et al. [79].

### Genetic model

Let *y*_*i*_ be phenotypic observation of the *i*th accessions (parent or F_1_) in the above partial NCII design. The genetic model for *y*_*i*_ is expressed as

$$y_{i}=\mu +\sum_{j}^{m}\left(x_{ij}a_{j}+z_{ij}d_{j}\right)+\sum_{j=1}^{m-1}\sum_{k=j+1}^{m}\left(x_{ij}x_{ik}{\left(aa\right)}_{jk}+x_{ij}z_{ik}{\left(ad\right)}_{jk}+z_{ij}x_{ik}{\left(da\right)}_{jk}+z_{ij}z_{ik}{\left(dd\right)}_{jk}\right)+\epsilon_{i}$$ (1)

where *μ* is the population mean; *m* is the number of putative QTL; *a*_*j*_ and *d*_*j*_ are the additive and dominant effects of the *j*th QTL (*j* = 1,∙∙∙,*m*), respectively; *x*_*ij*_ and *z*_*ij*_ are the dummy variables of the *i*th individual for *a*_*j*_ and *d*_*j*_, respectively; (*aa*)_*jk*_, (*ad*)_*jk*_, (*da*)_*jk*_ and (*dd*)_*jk*_ are *aa*, *ad*, *da*, and *dd* interaction effects between the *j*th and *k*th QTL (*j* = 1,∙∙∙,*m*−1;*k* > *j*), respectively; and *ε* is residual error with a *N*(0,*σ* ^2^) distribution.

For the sake of clarity of notation, we redefine the design matrix and the regression coefficients as follows. Let **Y** = (*y*_1_, *y*_2_, ∙∙∙, *y*_*n*_)^*T*^, **β** = *μ* and **X** = (1, 1, ∙∙∙, 1)^*T*^; **γ** is the main and interacted effects, and **Z** is the dummy variable for **γ**. The above model is now rewritten as

Y=Xβ+Zγ+ϵ (2)

### Parameter estimation by empirical Bayesian

There are several methods available in the estimation of parameters in model (2), e.g., penalized maximum likelihood [9,82], empirical Bayesian [10], hierarchical generalized linear model [83,84]. Here we adopt empirical Bayesian, for technical detail the reader is referred to the original study of Xu [10]. The method is briefly described here.

The parameters **β** and *σ* ^2^ are always included in the model, the uniform prior is assigned to the two parameters: *P*(**β**) ∝ 1 and *P*(*σ* ^2^) ∝ 1. We adopt the normal prior for each of the genetic effects (*γ*_*k*_) in model (2): $P(\gamma_{k})\propto N(0,\sigma_{k}^{2})$. The scaled inverse *χ* ^2^ prior distribution is further assigned to $\sigma_{k}^{2}$: $P(\sigma_{k}^{2})=\text{Inv-}\chi^{2}(\sigma_{k}^{2}|\tau ,\omega )\propto {\left(\sigma_{k}^{2}\right)}^{-\frac{\tau +2}{2}}\exp\left(-\omega /2\sigma_{k}^{2}\right)$. Clearly, **Y** in model (2) follows a multivariate normal distribution with mean *μ* = **Xβ** and variance-covariance $V=\sum Z_{k}Z_{k}{}^{T}\sigma_{k}^{2}+I\sigma^{2}$. Let θ = (**β**, **γ**, *σ* ^2^). Therefore, the main steps for parameter estimation are described as below.

Step (0): Let *ξ* = (*τ*,*ω*) = (0,0), $\hat{\beta }={\left(X^{T}X\right)}^{-1}X^{T}Y$, ${\hat{\sigma }}^{2}={\left(Y-X\hat{\beta }\right)}^{T}\left(Y-X\hat{\beta }\right)/n$, and *γ*_*k*_ and $\sigma_{k}^{2}$ were initialized (*k* = 1,2,∙∙∙,2*m* ^2^);
Steps (1): Using $E\left(\gamma_{k}\right)=\sigma_{k}^{2}Z_{k}^{T}V^{-1}\left(Y-X\beta \right)$ and $\mathrm{var}\left(\gamma_{k}\right)=I\sigma_{k}^{2}-\sigma_{k}^{2}Z_{k}^{T}V^{-1}Z_{k}\sigma_{k}^{2}$, $E\left(\gamma_{k}^{T}\gamma_{k}\right)$ was estimated by $E\left(\gamma_{k}^{T}\right)E\left(\gamma_{k}\right)+tr[\mathrm{var}\left(\gamma_{k}\right)]$. This is the E-step;
Step (2): Update **β**, *σ* ^2^ and $\sigma_{k}^{2}$: $\sigma_{k}^{2}=\left[E\left(\gamma_{k}^{T}\gamma_{k}\right)+\omega \right]/\left(\tau +2+1\right)$, **β** = (**X** ^*T*^ **V** ^−1^ **X**) ^−1^ **X** ^*T*^ **V** ^−1^ **Y**, and $\sigma^{2}={\left(Y-X\beta \right)}^{T}\left[Y-X\beta -\sum^{m}Z_{k}E\left(\gamma_{k}\right)\right]/n$. This is the M-step;
Step (3): Repeat the E-step and the M-step until convergence is reached.

### Likelihood ratio test

In this study, a two-stage approach was adopted to conduct significance test of each effect. First, empirical Bayesian was used to select the significant effects in model (2). Then, all the selected effects were tested by using likelihood ratio test. For technical detail the reader is referred to the original study of Zhang & Xu [9] and Lü et al. [14]. For simplicity, the critical LOD score for declaring a significant effect at the 0.05 level was set at 2.0.

### High throughput QTL-effect screening

A full genetic model should include potential pair-wise interaction effects of all loci. If the number of main and interacted effects is 10 times less than sample size, empirical Bayesian works well. Note that the model is saturated quickly as the number of loci increases. For example, in this study the number of SSR marker loci is 205, the numbers of potential main and interacted effects are 410 and 83,640, respectively. Therefore, a variable selection technique is usually considered to exclude those interactions with negligible effects. The procedures and steps were described as below.

1. **Test of independence between effect and trait.** First, extreme phenotypic individuals, 10% highest and 10% lowest, were selected from the mapping population. Then, contingency table was constructed based on the extreme individual and marker genotype. Third, *χ* ^2^ test of independence was conducted to test whether the targeted marker was associated with the trait. Finally, 100 main effects or 100 epistatic effects, with the minimum P-values, were selected to enter the next step. This method is BSA;
2. **Empirical Bayesian estimation and likelihood ratio test.** All the main or interacted effects selected in the first step were included in one genetic model and estimated by empirical Bayesian. Among these effects, the effects with non-zero estimates were remained. Likelihood ratio test was used to determine whether these effects were significantly associated with the trait. The critical LOD value was set at 2.0.
3. **Correction of trait phenotype using the significantly associated effects.** The corrected phenotypes for all the individual were $y_{i}^{\prime }=y_{i}-Wb$, where **b** was vector of effects for the significantly associated markers, and **W** was the designed matrix for *b*;
4. **Repeat the step (1) to (3) until no more additional significantly associated effects were detected**;
5. **Empirical Bayesian analysis for selected main and epistatic effects.** All the significantly main and interacted effects were included one genetic model and estimates by empirical Bayesian.

The software for parameter estimation is available as S1 Software.

### Hybrid prediction (HP)

In hybrid breeding, elite parents were predicted from general combining ability (GCA) and elite parental combinations were predicted from specific combining ability (SCA). In NCII mating design, both GCA for each parent and SCA for each parental combination could be calculated. In a partial NCII mating design, however, some F_1_ hybrids were missing so that GCA and SCA were not calculated. Using the information of QTL detected above, these missing values could be predicted. Once all the information was obtained, SCA and GCA could be calculated. Therefore, elite parents and parental combinations could be predicted.

### Monte Carlo simulation design

We performed three simulation experiments in this study. In the first simulation experiment, the effect of QTL heritability on the new method was assessed. The QTL size ($h_{i}^{2}$), being the proportion of total phenotypic variance explained by the QTL, was 0.02, 0.05 and 0.08, respectively. In each case, two additive, two dominant, one *aa*, one *ad*, one *da*, and one *dd* QTL were simulated. The genetic variance of the *i*th QTL, $\sigma_{i(G)}^{2}$, was calculated from $\sigma_{i(G)}^{2}=h_{i}^{2}\sigma^{2}/\left(1-\sum h_{i}^{2}\right)$, where residual variance *σ* ^2^ = 1. The allelic effects were calculated by relating $\sigma_{i(G)}^{2}$ to the allelic frequencies and effects. All the QTL were overlapped with the markers and listed in Table 3. All the genotypes of 441 parents and 284 F_1_ hybrids in the partial NCII were exactly same as those in real data analysis in this study. The simulated phenotypic value of each parent or F_1_ hybrid was the sum of the corresponding QTL genotypic values and residual error, with an assumed normal distribution. Each simulation run consisted of 100 replicates. For each simulated QTL, we counted the samples in which the LOD statistic surpassed 2.0. The ratio of the number of such samples to the total number of replicates represented the empirical power of this QTL. The FPR was calculated as the ratio of the number of false positive effects to the total number of zero effects. We also calculated absolute bias between estimated and true effects of each QTL in each sample. Therefore, the average and standard deviation of absolute biases across 100 replicates could be obtained. In the second simulation experiment, we evaluated the effect of sample size on the new method by letting the sample size be set as 400 (80 restorer lines + 160 sterile lines + 160 F_1_ hybrids), 500 (100 + 200 + 200) and 600 (120 + 240 + 240). All the QTL sizes were 0.05. Other parameters were the same as those in the first simulation experiment. In the third simulation experiment, we investigated the effect of population structure on the new method by letting the population structure be set as all the parents (300 restorer lines + 300 sterile lines), parents + F_1_ (150 restorer lines + 150 sterile lines + 300 F_1_ hybrids) and all the F_1_ (600 F_1_ hybrids). Other parameters were the same as those in the second simulation experiment.

**Table 3:** Parameter setupsin the Monte Carlo simulation studies.

Parameter setup	Case		
	1	2	3
Number of QTL	8 QTL (same setup)		
QTL type	2 additive, 2 dominant, 1 additive-by-additive, 1 additive-by-dominant, 1 dominant-by-additive, 1 dominant-by-dominant (same setup)		
QTL position (marker)	CB10597C, Bo3b, Ra2E12, CB10427A; MR049D × BnGMS439A, Ra2-G08A × Ra3-E05C, Bn1b × CB10431A, CB10036A × CB10045A (same setup)		
QTL size (%)	2, 5, 8	5	8
sample size	725	400, 500, 600	600
mapping population	Parents+F_1_	Parents+F_1_	all the parents, parents+F_1_, all the F_1_ hybrids

## Supporting Information

S1 Table Effect of QTL heritability on mapping QTL in NCII mating design. (DOCX)

S2 Table Effect of sample size on mapping QTL in NCII mating design. (DOCX)

S3 Table Effect of population structure on mapping QTL in NCII mating design. (DOCX)

S1 Software Software for mapping interacted QTL in partial NCII design. To first install JAVA Runtime Environment (jdk-7u71-windows-x64.exe) by default install directory, and to then install Maltlab Runtime Environment (R2014b (8.4) For Windows. The readers may download the first file at http://www.oracle.com/technetwork/java/javase/downloads/jdk7-downloads-1880260.html and the second file at http://cn.mathworks.com/products/compiler/mcr/index.html, respectively. If the readers want to use another jdk version, please change the corresponding content in the run.bat file. EMS memory size in the running computer is more than 8 G.
(ZIP)
