# Correction: Interacted QTL Mapping in Partial NCII Design Provides Evidences for Breeding by Design

**DOI:** 10.1371/journal.pone.0127131

**Published:** 2015-04-29

**Authors:** The PLOS ONE Staff

The first and last names for the second and the fifth authors were inadvertently switched. The first name appears as the last name and the last name appears as the first name. Zhao Xinwang should be Xinwang Zhao and Tu Jinxing should be Jinxing Tu. The correct citation is: Bu SH, Zhao X, Yi C, Wen J, Tu J, Zhang YM (2015) Interacted QTL Mapping in Partial NCII Design Provides Evidences for Breeding by Design. PLoS ONE 10(3): e0121034. doi:10.1371/journal.pone.0121034. The publisher apologizes for this error.

There are errors in the author contributions. The initials for Xinwang Zhao should be XZ and the initials for Jinxing Tu should be JT.

An additional email address for the corresponding author is missing. The additional email address is: soyzhang@mail.hzau.edu.cn.
