# Supplementary material for: Interacted QTL Mapping in Partial NCII Design Provides Evidences for Breeding by Design
Source: PLoS One. 2015 Mar 30;10(3):e0121034. doi: 10.1371/journal.pone.0121034 (PMC4379165; doi:10.1371/journal.pone.0121034)
Supplement: S1 Table — (DOCX) [file pone.0121034.s001.docx]

**S1_Table. Effect of QTL heritability on mapping QTL in NCII mating design**

| **r^2^** | **Parameter** | | |  | **Estimate** | | |
| --- | --- | --- | --- | --- | --- | --- | --- |
|  | **QTL** | **Type** | **Position (marker)** |  | **Power(%)** | **Absolute bias ± SD** | **FPR(‰)** |
| 2% | 1 | additive (a) | CB10597C |  | 83 | 0.0372 ± 0.0281 | 0.209 |
|  | 2 | a | Bo3b |  | 69 | 0.0399 ± 0.0342 |  |
|  | 3 | dominant (d) | Ra2E12 |  | 59 | 0.1234 ± 0.0870 |  |
|  | 4 | d | CB10427A |  | 61 | 0.1178 ± 0.0932 |  |
|  | 5 | additive-by-additive (aa) | MR049D × BnGMS439A |  | 44 | 0.0286 ± 0.0202 |  |
|  | 6 | additive-by-dominant (ad) | Ra2-G08A × Ra3-E05C |  | 31 | 0.1093 ± 0.0776 |  |
|  | 7 | dominant-by-additive (da) | Bn1b × CB10431A |  | 19 | 0.0930 ± 0.0730 |  |
|  | 8 | dominant-by-dominant (dd) | CB10036A × CB10045A |  | 8 | 0.1133 ± 0.1882 |  |
| 5% | 1 | a | CB10597C |  | 100 | 0.0417 ± 0.0320 | 0.191 |
|  | 2 | a | Bo3b |  | 100 | 0.0428 ± 0.0332 |  |
|  | 3 | d | Ra2E12 |  | 91 | 0.1128 ± 0.0872 |  |
|  | 4 | d | CB10427A |  | 100 | 0.1581 ± 0.1238 |  |
|  | 5 | aa | MR049D × BnGMS439A |  | 100 | 0.0392 ± 0.0304 |  |
|  | 6 | ad | Ra2-G08A × Ra3-E05C |  | 95 | 0.1525 ± 0.1108 |  |
|  | 7 | da | Bn1b × CB10431A |  | 91 | 0.1287 ± 0.0937 |  |
|  | 8 | dd | CB10036A × CB10045A |  | 66 | 0.1548 ± 0.1169 |  |
| 8% | 1 | a | CB10597C |  | 100 | 0.0448 ± 0.0313 | 0.205 |
|  | 2 | a | Bo3b |  | 100 | 0.0443 ± 0.0331 |  |
|  | 3 | d | Ra2E12 |  | 100 | 0.1221 ± 0.0916 |  |
|  | 4 | d | CB10427A |  | 100 | 0.1874 ± 0.1537 |  |
|  | 5 | aa | MR049D × BnGMS439A |  | 100 | 0.0453 ± 0.0326 |  |
|  | 6 | ad | Ra2-G08A × Ra3-E05C |  | 100 | 0.1616 ± 0.1171 |  |
|  | 7 | da | Bn1b × CB10431A |  | 100 | 0.1544 ± 0.1048 |  |
|  | 8 | dd | CB10036A × CB10045A |  | 94 | 0.1889 ± 0.1665 |  |
