# Supplementary material for: Interacted QTL Mapping in Partial NCII Design Provides Evidences for Breeding by Design
Source: PLoS One. 2015 Mar 30;10(3):e0121034. doi: 10.1371/journal.pone.0121034 (PMC4379165; doi:10.1371/journal.pone.0121034)
Supplement: S2 Table — (DOCX) [file pone.0121034.s002.docx]

**S2_Table. Effect of sample size on mapping QTL in NCII mating design**

| **Sample size** | **Parameter** | | |  | **Estimate** | | |
| --- | --- | --- | --- | --- | --- | --- | --- |
|  | **QTL** | **Type** | **Position (marker)** |  | **Power (%)** | **Absolute bias ± SD** | **FPR(‰)** |
| 400 | 1 | additive (a) | CB10597C |  | 97 | 0.0520 ± 0.0349 | 0.196 |
|  | 2 | a | Bo3b |  | 96 | 0.0589 ± 0.0461 |  |
|  | 3 | dominant (d) | Ra2E12 |  | 78 | 0.1790 ± 0.1383 |  |
|  | 4 | d | CB10427A |  | 88 | 0.2382 ± 0.2071 |  |
|  | 5 | additive-by-additive (aa) | MR049D × BnGMS439A |  | 80 | 0.0509 ± 0.0376 |  |
|  | 6 | additive-by-dominant (ad) | Ra2-G08A × Ra3-E05C |  | 29 | 0.1462 ± 0.1399 |  |
|  | 7 | dominant-by-additive (da) | Bn1b × CB10431A |  | 37 | 0.2010 ± 0.1450 |  |
|  | 8 | dominant-by-dominant (dd) | CB10036A × CB10045A |  | 9 | 0.1700 ± 0.1292 |  |
| 500 | 1 | a | CB10597C |  | 99 | 0.0470 ± 0.0344 | 0.206 |
|  | 2 | a | Bo3b |  | 100 | 0.0554 ± 0.0407 |  |
|  | 3 | d | Ra2E12 |  | 81 | 0.1582 ± 0.1067 |  |
|  | 4 | d | CB10427A |  | 96 | 0.2468 ± 0.1777 |  |
|  | 5 | aa | MR049D × BnGMS439A |  | 90 | 0.0539 ± 0.0383 |  |
|  | 6 | ad | Ra2-G08A × Ra3-E05C |  | 57 | 0.1744 ± 0.1237 |  |
|  | 7 | da | Bn1b × CB10431A |  | 61 | 0.1645 ± 0.1115 |  |
|  | 8 | dd | CB10036A × CB10045A |  | 31 | 0.1773 ± 0.1294 |  |
| 600 | 1 | a | CB10597C |  | 100 | 0.0474 ± 0.0319 | 0.208 |
|  | 2 | a | Bo3b |  | 99 | 0.0552 ± 0.0362 |  |
|  | 3 | d | Ra2E12 |  | 84 | 0.1551 ± 0.1043 |  |
|  | 4 | d | CB10427A |  | 97 | 0.2149 ± 0.1633 |  |
|  | 5 | aa | MR049D × BnGMS439A |  | 99 | 0.0528 ± 0.0429 |  |
|  | 6 | ad | Ra2-G08A × Ra3-E05C |  | 89 | 0.1500 ± 0.1087 |  |
|  | 7 | da | Bn1b × CB10431A |  | 82 | 0.1333 ± 0.0915 |  |
|  | 8 | dd | CB10036A × CB10045A |  | 46 | 0.1783 ± 0.1127 |  |
