# Supplementary material for: Interacted QTL Mapping in Partial NCII Design Provides Evidences for Breeding by Design
Source: PLoS One. 2015 Mar 30;10(3):e0121034. doi: 10.1371/journal.pone.0121034 (PMC4379165; doi:10.1371/journal.pone.0121034)
Supplement: S3 Table — (DOCX) [file pone.0121034.s003.docx]

**S3_Table. Effect of population structure on mapping QTL in NCII mating design**

| **Case** | **QTL** | **Parameter** | | |  | **Estimate** | | |
| --- | --- | --- | --- | --- | --- | --- | --- | --- |
|  |  | **Type** | **Position (marker)** | **P_1_ :P_2_:F_1_** |  | **Power (%)** | **Absolute bias ± SD** | **FPR(‰)** |
| 1 | 1 | additive (a) | CB10597C | 1:1:0 |  | 100 | 0.0380 ± 0.0318 | 0.067 |
|  | 2 | a | Bo3b |  |  | 99 | 0.0327 ± 0.0241 |  |
|  | 3 | dominant (d) | Ra2E12 |  |  | - | - |  |
|  | 4 | d | CB10427A |  |  | - | - |  |
|  | 5 | additive-by-additive (aa) | MR049D × BnGMS439A |  |  | 98 | 0.0390 ± 0.0273 |  |
|  | 6 | additive-by-dominant (ad) | Ra2-G08A × Ra3-E05C |  |  | - | - |  |
|  | 7 | dominant-by-additive (da) | Bn1b × CB10431A |  |  | - | - |  |
|  | 8 | dominant-by-dominant (dd) | CB10036A × CB10045A |  |  | - | - |  |
| 2 | 1 | a | CB10597C | 1:1:2 |  | 100 | 0.0428 ± 0.0360 | 0.195 |
|  | 2 | a | Bo3b |  |  | 99 | 0.0480 ± 0.0388 |  |
|  | 3 | d | Ra2E12 |  |  | 91 | 0.1144 ± 0.0885 |  |
|  | 4 | d | CB10427A |  |  | 98 | 0.2023 ± 0.1585 |  |
|  | 5 | aa | MR049D × BnGMS439A |  |  | 95 | 0.0463 ± 0.0376 |  |
|  | 6 | ad | Ra2-G08A × Ra3-E05C |  |  | 86 | 0.1288 ± 0.0902 |  |
|  | 7 | da | Bn1b × CB10431A |  |  | 81 | 0.1163 ± 0.0998 |  |
|  | 8 | dd | CB10036A × CB10045A |  |  | 49 | 0.1168 ± 0.0931 |  |
| 3 | 1 | a | CB10597C | 0:0:1 |  | 100 | 0.0575 ± 0.0438 | 0.108 |
|  | 2 | a | Bo3b |  |  | 98 | 0.0642 ± 0.0510 |  |
|  | 3 | d | Ra2E12 |  |  | 91 | 0.0992 ± 0.0730 |  |
|  | 4 | d | CB10427A |  |  | 99 | 0.1525 ± 0.1165 |  |
|  | 5 | aa | MR049D × BnGMS439A |  |  | 59 | 0.0673 ± 0.0588 |  |
|  | 6 | ad | Ra2-G08A × Ra3-E05C |  |  | 45 | 0.0975 ± 0.0635 |  |
|  | 7 | da | Bn1b × CB10431A |  |  | 48 | 0.0822 ± 0.0718 |  |
|  | 8 | dd | CB10036A × CB10045A |  |  | 48 | 0.1088 ± 0.0762 |  |
